# Inducible EphA4 knockout causes motor deficits in young mice and is not protective in the SOD1^G93A^ mouse model of ALS

**DOI:** 10.1038/s41598-020-72723-y

**Published:** 2020-09-24

**Authors:** Sara L. Dominguez, Timothy Earr, Michelle Dourado, Hai Ngu, William J. Meilandt, Jesse E. Hanson

**Affiliations:** 1 Genentech, Neuroscience, South San Francisco, 94080 USA; 2 Genentech, Pathology, South San Francisco, 94080 USA

**Keywords:** Neuromuscular junction, Spinal cord, Neurological disorders, Motor neuron disease, Amyotrophic lateral sclerosis

## Abstract

Amyotrophic lateral sclerosis (ALS) is a neurodegenerative disease characterized by motor neuron loss that ultimately leads to fatal paralysis. Reducing levels or function of the tyrosine kinase, ephrin type-A receptor 4 (EphA4), has been suggested as a potential approach for slowing disease progression in ALS. Because EphA4 plays roles in embryonic nervous system development, study of constitutive knockout (KO) of EphA4 in mice is limited due to confounding phenotypes with homozygous knockout. We used a tamoxifen-inducible EphA4 conditional KO mouse to achieve strong reduction of EphA4 levels in postnatal mice to test for protective effects in the SOD1^G93A^ model of ALS. We found that EphA4 KO in young mice, but not older adult mice, causes defects in muscle function, consistent with a prolonged postnatal role for EphA4 in adolescent muscle growth. When testing the effects of inducible EphA4 KO at different timepoints in SOD1^G93A^ mice, we found no benefits on motor function or disease pathology, including muscle denervation and motor neuron loss. Our results demonstrate deleterious effects of reducing EphA4 levels in juvenile mice and do not provide support for the hypothesis that widespread EphA4 reduction is beneficial in the SOD1^G93A^ mouse model of ALS.

## Introduction

Amyotrophic lateral sclerosis (ALS) is a neurodegenerative disease affecting upper and lower motor neurons of the cortex and spinal cord, respectively, leading to paralysis and death. The prevalence of ALS in the United States population is 5 per 100,000 with a majority of patients dying within 5 years of diagnosis. To date, there is no cure for ALS and only two treatments approved by the U.S. Food and Drug Administration, Riluzole, which has been shown to increase life expectancy by 3–6 months, and Edaravone, which has been shown to reduce the decline of daily function when given to patients in the early stage of disease. Since only 10 percent of ALS cases are driven by known genetic mutations^1^, such as *SOD1* or *C9orf72,* therapeutic interventions directly targeting and correcting for these mutations may prove beneficial, but may not be effective in the majority of sporadic cases. Identification of genes that modify the progression of the disease could point to therapeutic targets with the potential to benefit a broader range of patients. One such target, EphA4, was identified as a disease modifying gene in a screen using zebrafish ALS models^2^. That study also found benefits of reducing EphA4 in rodent models of ALS, including preserved spinal cord motor neurons, increased neuromuscular junction (NMJ) innervation, and improved survival with heterozygous EphA4 KO in the superoxide dismutase 1 (SOD1^G93A^) mouse model^2^.

The identification of a protective role for EphA4 reduction in ALS models, along with evidence suggesting EphA4 inhibition could be protective in mouse models of Alzheimer’s disease, spinal cord injury, and stroke^3–6^, prompted interest in developing EphA4 inhibitors as a potential therapeutic strategy for treating neurodegeneration. Subsequent work designing and characterizing EphA4 targeting agents identified a compound (123C4) that prolonged survival in SOD1^G93A^ mice^7^. However, that compound was found to act as an EphA4 agonist in cellular assays, raising questions about whether the in vivo benefits were due to EphA4 activation or EphA4 down-regulation following agonist binding.

Consistent with the original notion that reducing EphA4 is beneficial, a recent study found that treatment with an EphA4 inhibitor (EphA4 ectodomain fused to Fc) starting at 35 days of age improved functional performance in SOD1^G93A^ mice^8^. That study also found that heterozygous motor neuron-specific EphA4 knockout preserved motor neurons and neuromuscular junctions, improved functional performance, and delayed disease onset in SOD1^G93A^ mice. On the other hand, a different study found that CNS reduction of EphA4 levels with antisense oligonucleotides delivered directly to the central nervous system starting at 49 days of age had no benefit for motor function or survival in two ALS models, including SOD1^G93A^ mice^9^. Similarly, recent work using inducible KO of EphA4 found that reduction of global EphA4 or motoneuron EphA4 starting at 60 days of age did not modify disease course or improve survival in SOD1^G93A^ mice^10^.

The varying results across previous studies could be contributed to the differences in the timing and level of EphA4 inhibition achieved in each study. Because EphA4 is repulsive to axon growth and plays roles in spinal circuit formation during early development^11^, constitutive homozygous KO results in abnormalities in motor function, limiting previous studies to examining the effects of heterozygous KO (i.e., 50% reduction in EphA4) in SOD1^G93A^ mice. On the other hand, studies using inducible KO or pharmacological inhibition to minimize developmental deficits have typically started intervention at later timepoints in SOD1^G93A^ mice. To better understand the potential of EphA4 inhibition at different stages of disease progression in SOD1^G93A^ mice, we used conditional EphA4 KO mice with tamoxifen-induced cre expression beginning at 28 or 49 days of age. Our results reveal a role for EphA4 in normal muscle function that persists in juvenile and adult mice. At the same time, we find no evidence for a protective role of EphA4 KO in SOD1^G93A^ mice.

## Results

### Robust EphA4 knockdown can be achieved in brain and spinal cord of tamoxifen-inducible conditional KO mice

**Figure 1:**
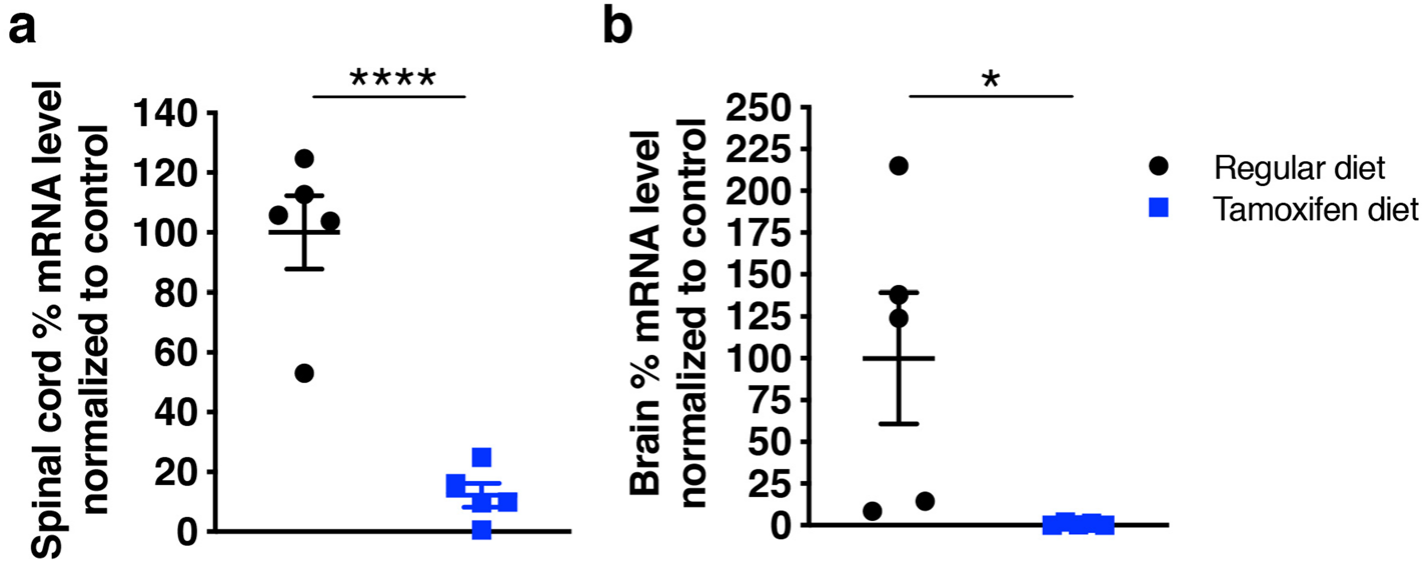
Reduction of EphA4 in brain and spinal cord in homozygous floxed EphA4 mice expressing tamoxifen-inducible cre after tamoxifen treatment. **(a)** Spinal cord EphA4 levels for mice placed on tamoxifen diet compared to mice on control diet (p < 0.0001) (n = 5/group). **(b)** Brain Epha4 mRNA levels (p < 0.05) (n = 5/group).

To bypass the potential for detrimental effects of EphA4 KO on axon guidance in early development^11^, we crossed floxed EphA4 conditional KO mice to tamoxifen-inducible CAG Cre mice to allow for postnatal removal of EphA4 with tamoxifen treatment. To test the level of EphA4 depletion that could be achieved with tamoxifen induction, adult EphA4 Cre + mice were placed on either tamoxifen or control diet for 4 weeks, after which mRNA levels were tested via quantitative polymerase chain reaction (qPCR). In spinal cord, the tamoxifen-fed group showed an average of 87% reduction of EphA4 mRNA (Fig. 1a) and near complete reduction was observed in the brain (Fig. 1b). Thus, this approach leads to robust depletion of EphA4.

### EphA4 KO causes abnormal muscle development and function when induced in juvenile mice and exacerbates deficits in SOD1^G93A^ mice

**Figure 2:**
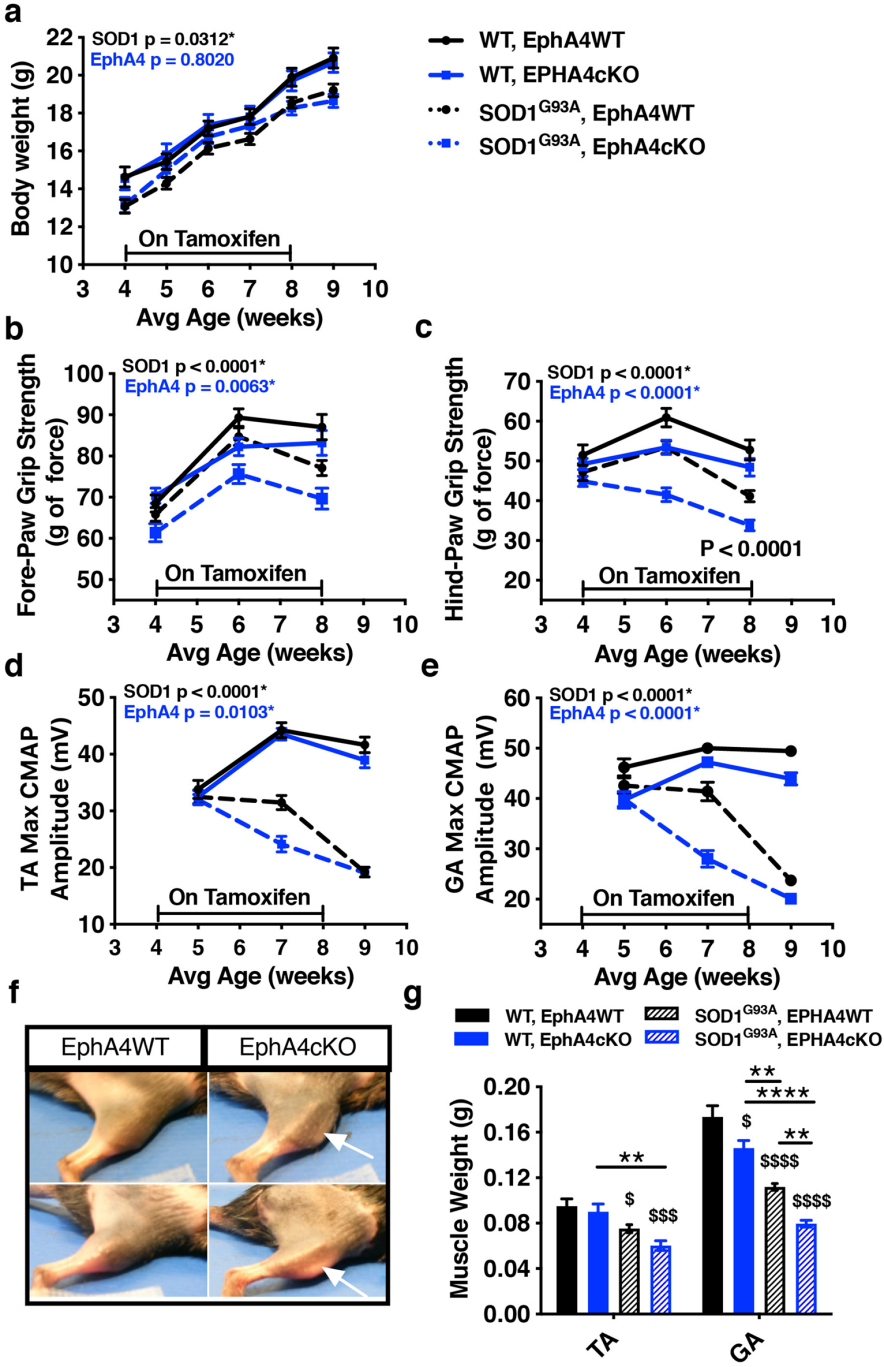
EphA4 KO is detrimental to muscle growth and function when induced starting at 4 weeks of age and exacerbates deficits in SOD1^G93A^ mice. **(a)** Reduced body weight in SOD1^G93A^ mice is unaffected by EphA4 KO. RMANOVA on body weight revealed significant effects of genotype (F (3, 58) = 3.16; p = 0.0312*), time (F (5, 54) = 411.33; p < 0.0001) and a genotype/time interaction (F (15, 168) = 2.83; p = 0.0006). Follow up analysis revealed effects of SOD1 genotype (F (1, 58) = 9.28; p = 0.0035) but not EPHA4 genotype. **(b–e)** Fore-paw and hind-paw grip strength (b,c) and TA and GA CMAP amplitude (d,e) are reduced in SOD1^G93A^ mice and impaired by EphA4 KO. RMANOVAs (b-e) all revealed significant effects of genotype, time and genotype/time interactions. **(b)** Significant effects of SOD1 genotype (F(1, 58) = 19.17; P < 0.0001) and EphA4 genotype (F(1, 58) = 8.05; p = 0.0063) on fore-paw strength. **(c)** Significant effects of SOD1 genotype (F(1, 58) = 30.18; p < 0.0001) and EphA4 genotype (F(1, 58) = 16.52; p < 0.0001) on hind-paw strength. **(d)** Significant effects of SOD1 genotype (F(1, 58) = 256.78; p < 0.0001) and EphA4 genotype (F(1, 58) = 7.04; p < 0.0103) on TA CMAP amplitude. **(e)** Significant effects of SOD1 genotype (F(1, 58) = 238.88; P < 0.0001) and EphA4 genotype (F(1, 58) = 43.4103; p < 0.0001) on GA CMAP amplitude. (n = 13–14 in WT, 17–18 in SOD1 groups). **(f, g)** TA and GA muscle weight are reduced by SOD1^G93A^ and EphA4 KO. **(f)** Images of right hind legs of EphA4WT and EphA4KO mice at 9 weeks of age. Arrows indicate regions of altered muscle growth in EphA4cKO mice. **(g)** One-way ANOVA revealed significant effects of genotype on TA (F(3, 31) = 9.487; p < 0.0001) and GA (F(3,32) = 50.30; p < 0.0001) muscle weight. Post hoc Tukey’s t-test, revealed a significant effect of SOD1 genotype on TA muscle weights and significant effects of SOD1 and EphA4 genotypes on GA muscle weight. (n = 8 in WT, 10 in SOD1 groups).

To test whether EphA4 KO is protective in SOD1^G93A^ mice, we bred cohorts of mice that all contained floxed EphA4 alleles, and varied in the expression of the SOD1^G93A^ vs WT allele and were negative or positive for tamoxifen-inducible cre. All mice were then treated with tamoxifen at the same time. Because conditional EphA4 KO only occurs in the cre positive mice, we refer to the cre negative mice as EphA4WT and the cre positive mice as EphA4cKO. For the initial study, all mice were placed on tamoxifen diet at 4 weeks of age for 4 weeks and then returned to normal diet. Body weight was measured weekly and, as expected, the SOD1^G93A^ groups weighed slightly less than their WT littermates, while weights were not altered by EphA4 KO (Fig. 2a). To determine whether EphA4 KO alters functional decline in SOD1^G93A^ mice, we tested fore-paw and hind-paw grip strength and performed compound muscle action potential recordings (CMAP) in the tibialis anterior (TA) and gastrocnemius (GA) muscles. These measures were performed at the beginning of the study and then repeated at two-week intervals after initiating KO. During the study, SOD1^G93A^ mice exhibited declines in grip strength and CMAP amplitude relative to WT mice (Fig. 2b–e). At the same time, EphA4 KO was not protective in the SOD1^G93A^ mice but rather significantly reduced grip strength and CMAP amplitude independent of SOD1 genotype (Fig. 2b–e). Consistent with a detrimental role for EphA4 KO, hind limb muscle growth was also impacted by EphA4 KO when tissue was collected at the end of the study. Muscle weight was reduced in WT EphA4cKO mice and reduced muscle weight in SOD1^G93A^ mice was exacerbated by EphA4cKO (Fig. 2f,g).

### Initiating KO at later ages lessens detrimental effects of EphA4 reduction on muscle function, but does not provide benefit in SOD1^G93A^ mice

**Figure 3:**
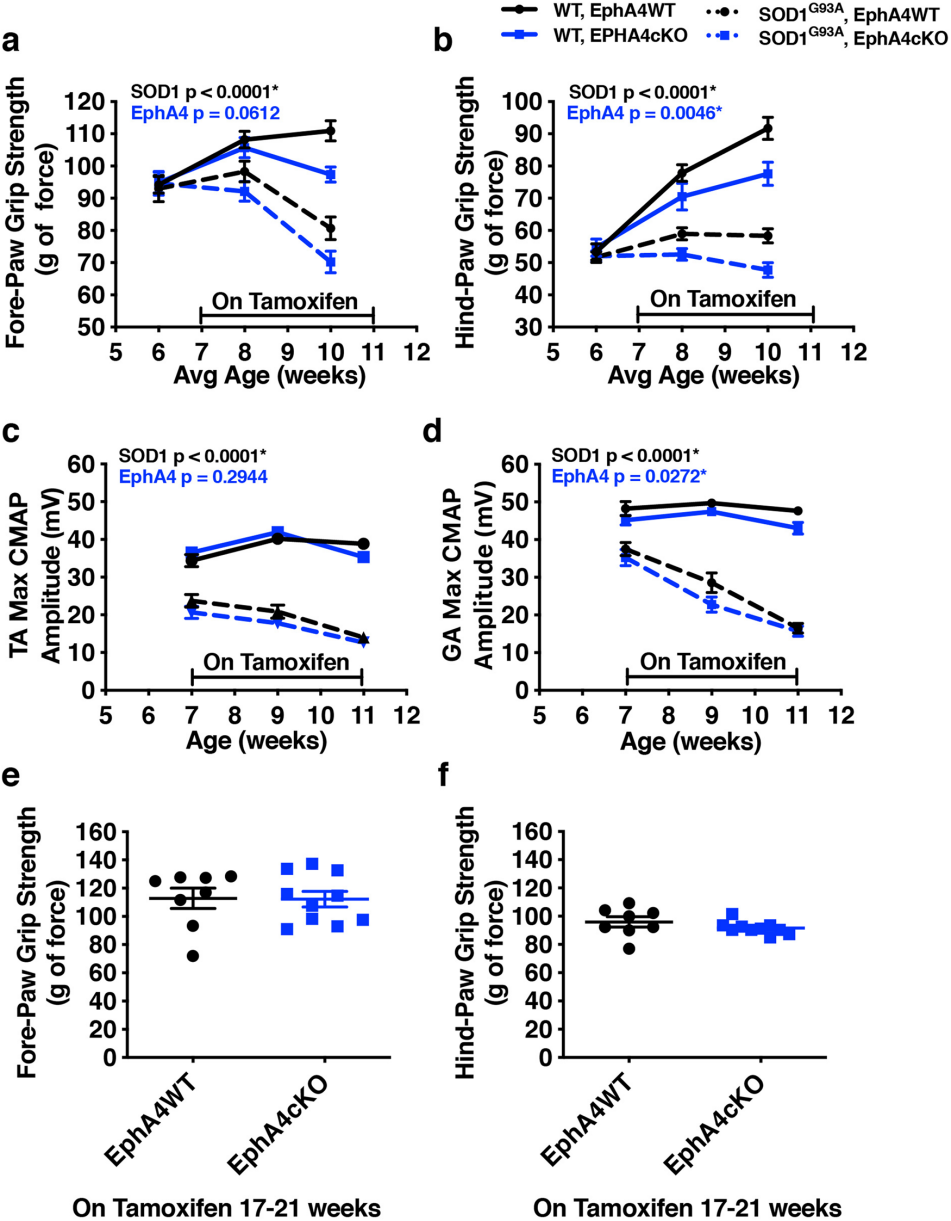
Initiating EphA4 KO at a later age has less detrimental effects on muscle function, but does not provide benefit in SOD1^G93A^ mice. **(a–d)** Grip strength **(a, b)** and CMAP amplitude **(c, d)** are reduced to a lesser extent in SOD1^G93A^ mice when Tamoxifen-induced EphA4 KO is initiated at 7 weeks of age. RMANOVAs **(a–d)** all revealed significant effects of genotype, time and genotype/time interactions. **(a)** Significant effects on fore-paw grip strength were observed for SOD1 genotype (F(1, 57) = 26.65; p < 0.0001) but not EphA4 genotype, however Tukey’s T-test show a significant effect of EphA4 genotype at the 10 week time point (p = 0.009). **(b)** Hind-paw strength was effected by both SOD1 (F(1, 58) = 70.02; p < 0.0001) and EphA4 genotypes (F(1,58) = 8.70; p = 0.0046). **(c)** TA muscle CMAP amplitude was significantly affected by SOD1 (F(1, 58) = 70.02; p < 0.0001) but not EphA4 genotype. **(d)** RMANOVA on CMAP amplitude in GA muscle revealed effects of both SOD1 (F(1, 58) = 225.32; p < 0.0001) and EphA4 genotype (F(1, 58) = 5.14; p = 0.0272). (n = 12–14 in WT groups, 18 in SOD1 groups, fig. **a–d**). **(e, f)** Detrimental effects of EphA4 are fully attenuated when KO is induced at 17–21 weeks of age in WT mice, but SOD1^G93A^ mice are end stage at this age range, precluding test of EphA4 KO effects in SOD1^G93A^ mice. **(e)** Unpaired t-test reveals no difference in fore-paw grip strength between EphA4WT and EphA4cKO mice at 22 weeks of age **(f)** Unpaired t-test reveals no difference in hind-paw grip strength between EphA4WT and EphA4cKO mice at 22 weeks of age. (n = 8 EphA4WT and 10 EphA4cKO, all females, fig **e, f**).

Given that the detrimental effects of EphA4 KO, when initiated at 4 weeks of age, occur even in WT mice, any roles for EphA4 that are specific to the context of the SOD1^G93A^ model are difficult to decipher. Therefore, we next tested whether the abnormal muscle phenotype observed in EphA4cKO mice was affected by the age at which EphA4 KO was initiated. In an attempt to avoid a possible developmental phenotype but to reduce EphA4 early enough to see potential benefits on SOD1^G93A^ pathology, we placed a cohort of mice on tamoxifen diet from 7–11 weeks of age. We found that EphA4 KO still had detrimental effects on the grip strength which were independent of SOD1 genotype (Fig. 3a,b). However, CMAP amplitude was not as significantly affected by EphA4 KO (Fig. 3c,d) indicating less severe muscle phenotypes when KO was induced at an older age. At the same time, as with the younger mice, there was no evidence for a beneficial effect of EphA4 KO in the older SOD1^G93A^ mice who showed strong declines in grip strength and CMAP amplitude relative to WT mice, regardless of EphA4 KO (Fig. 3a–d).

EphA4 protein expression in rodent skeletal muscle can persist in young adult animals before declining in older animals^12^. Therefore, to better understand the time course of the EphA4-dependence of muscle function we placed a cohort of mice on tamoxifen diet from 17 to 21 weeks of age. Because SOD1^G93A^ mice are close to end stage disease by 21 weeks, we were not able include SOD1^G93A^ groups in this cohort. In contrast to the younger groups of mice, we saw no adverse effect of EphA4 KO when initiated at 17 weeks of age (Fig. 3e,f). This indicates that muscle phenotypes can be avoided if KO is initiated in fully developed adult mice. Unfortunately, testing EphA4cKO for effects on functional decline in this aggressive SOD1^G93A^ model will always be confounded by this developmental phenotype, since treatment will most likely need to occur before muscle growth is complete.

### EphA4 KO does not protect against NMJ denervation or motor neuron loss in SOD1^G93A^ mice

**Figure 4:**
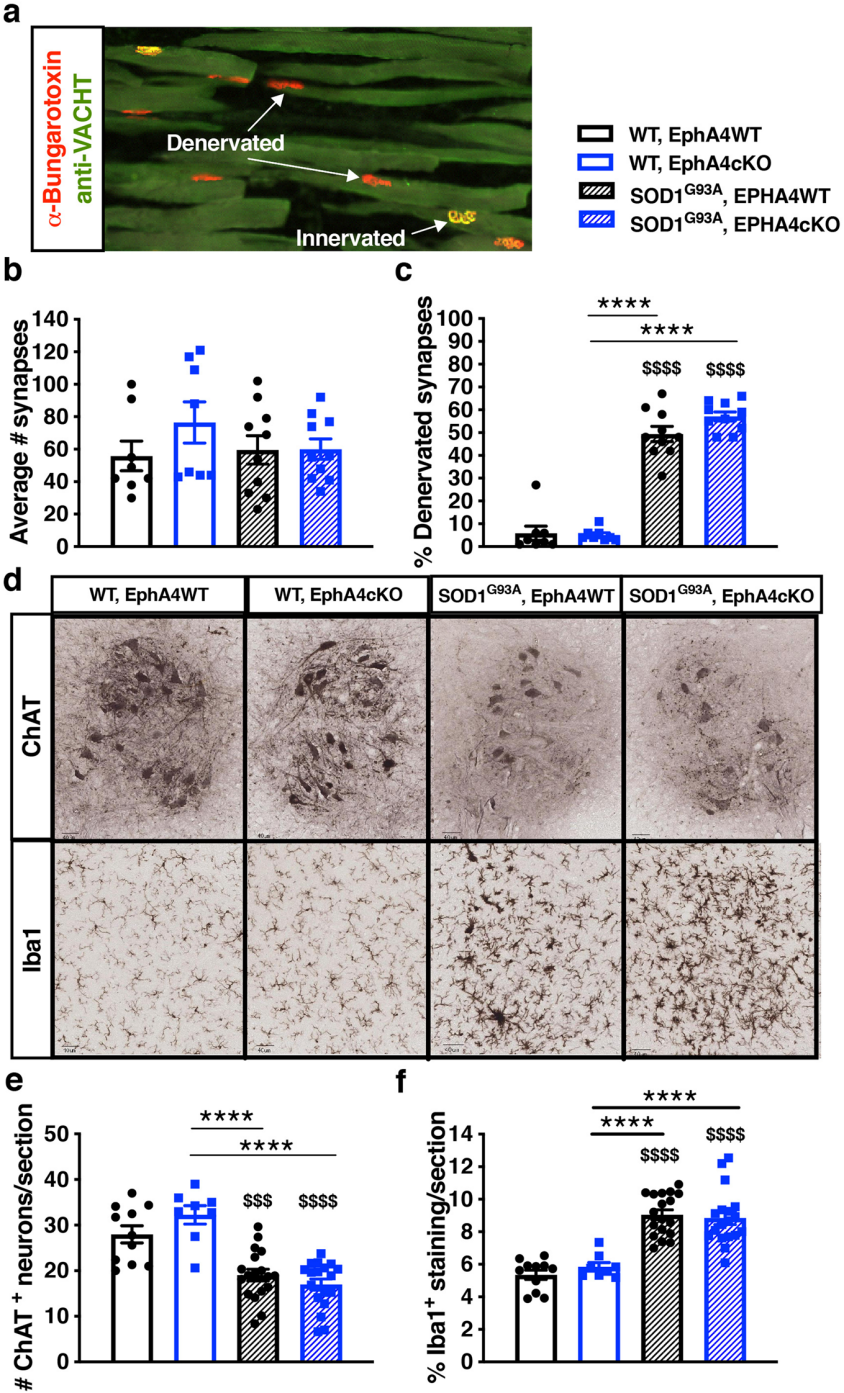
EphA4 KO does not protect against NMJ denervation, motor neuron loss or gliosis in SOD1^G93A^ mice. **(a–c)** The early phenotype of NMJ loss was examined in 9-week TA muscle tissue from the cohort of mice with EphA4 KO initiated at 4 weeks (Fig. 2). **(a)** Examples of innervated and denervated NMJs co-stained with anti-VACHT (green) and α-Bungarotoxin (red). **(b)** One-way ANOVA comparing total number of synapses found no differences between the four genotypes. (n = 8 in WT groups, 10 in SOD1 groups). **(c)** One-way ANOVA comparing % denervation found an overall significant effect of genotype (F(3, 32) = 112.8; p < 0.0001). Follow up with Tukey’s t-test, revealed a significant effect of SOD1 genotype but not EphA4 genotype. (n = 8 in WT groups, 10 in SOD1 groups). **(d–f)** The later phenotypes of spinal cord motor neuron loss and gliosis was examined in 15-week-old tissue from the cohort of mice with EphA4 KO initiated at 7 weeks (Fig. 3). **(d)** Sample images of ChAT stained motor neurons and Iba1 stained microglia in lumbar spinal cord in the different genotypes. **(e)** L3-L5 motor neuron counts analyzed by one-way ANOVA showed an overall genotype group effect (F(3, 51) = 19.17; p < 0.0001), with post-hoc Tukey’s t-test revealing significant effects of SOD1 but not EphA4 genotype. (n = 8–10 in WT groups, 18 in SOD1 groups). **(f)** One-way ANOVA comparing Iba1 stained microglia in lumbar spinal cord shows an overall effect across groups (F(3, 51) = 27.13; p < 0.001) with post-hoc Tukey’s t-test showing increased gliosis in the SOD1 groups but no changes when EphA4 is knocked down. (n = 8–10 in WT groups, 18 in SOD1 groups).

Although muscle function and growth were abnormal in mice when EphA4 KO was started early, it is still possible that EphA4 KO could have a protective effect on other aspects of SOD1^G93A^ disease pathology. To test this, we examined muscle innervation, which is an early phenotype, in the cohort of mice which underwent tamoxifen treatment from 4–8 weeks. To assess the number of NMJ synapses, we measured the total number of alpha-bungarotoxin positive endplates in tissue taken at 9 weeks of age. We saw no overall difference in the total number of synapses between the genotypes (Fig. 4a,b). Next, we looked at the percentage of denervated synapses using colocalization of the vesicular acetylcholine transporter (VAChT) which labels presynaptic terminals, and alpha-bungarotoxin positive endplates. As expected, the SOD1^G93A^ mice showed significant denervation relative to WT mice (Fig. 4a,c). At the same time, EphA4 KO did not change innervation in WT mice and did not prevent denervation in SOD1^G93A^ mice.

We next looked at the later phenotypes of motor neuron loss and gliosis in the lumbar spinal cord using the cohort of mice which underwent tamoxifen treatment from 7–11 weeks of age. We quantified both ChAT staining for motor neurons and Iba1 staining for microglia (Fig. 4d–f) at 15 weeks of age. While the density of ChAT positive neurons was decreased, and Iba1 positive microglia increased, in the SOD1^G93A^ mice, there was no effect of EphA4 KO on these phenotypes. These experiments indicated that neither the neurodegeneration or neuroinflammation seen in SOD1^G93A^ mice were altered by EphA4 KO.

## Discussion

Using conditional KO of EphA4 at various ages we determined that, aside from roles in motor neurons, EphA4 plays a role in muscle function that persists during postnatal muscle development in mice. At the same time, we observed no beneficial effects, only worsening of function in SOD1^G93A^ mice with EphA4 KO. Furthermore, EphA4 KO did not protect against motor neuron pathology in SOD1^G93A^ mice. Together, these results speak to challenges with the therapeutic time window in this model, while at the same time fail to support the hypothesis that EphA4 reduction is beneficial.

In addition to questions about the generalizability of the SOD1^G93A^ model to ALS pathophysiology that is not driven by SOD1 mutations, the aggressive nature of this model presents challenges for preclinical therapeutic experiments. This is illustrated by the overlap of the rapid decline in function and short survival of these mice with the age range that involves the prolonged developmental roles for EphA4 that we observe. In this regard, the more recently developed very low expressing SOD1^G93A^ transgenic model^13^ could be beneficial in future studies evaluating targets with both developmental roles and potential disease modifying effects. The prolonged survival and protracted motor neuron loss in that model should not only better mimic ALS disease course, but allow a longer and later window for therapeutic interventions. It should be noted that the translatability of the role for EphA4 in muscle development/maintenance that we see in mice to humans is unknown, and this role for EphA4 would not necessarily be relevant at the age that ALS patients are typically diagnosed.

Despite limitations with the SOD1^G93A^ model, our results raise significant doubts about the therapeutic hypothesis that reducing EphA4 is beneficial. Our failure to observe any benefits of EphA4 KO in grip strength, CMAP amplitude, NMJ denervation or motor neuron loss phenotypes, and worsening of some of these phenotypes in the SOD1^G93A^ model is consistent with other studies that failed to see benefits of postnatal reduction of EphA4 levels with antisense oligonucleotides^9^ or with inducible global or motoneuron EphA4 KO^10^. One potential limitation of our study is that we did not perform survival analysis. However, in our experience testing potential therapeutic manipulations in the SOD1^G93A^ model, we have never observed a survival benefit in the absence of benefits in histological or functional endpoints, and we and others have also sometimes failed to observe survival benefits even when benefits in the histological and functional endpoints have been observed^8,14–16^. Therefore, as EphA4 KO failed to rescue any phenotypes and had some detrimental effects in our study, we did not conduct a survival study.

As previous studies have found benefits with constitutive heterozygous EphA4 KO either globally^2^ or restricted to motor neurons^8^, this raises the possibility that the level of EphA4 reduction might need to be intermediate to be beneficial, and that higher levels of reduction, as in our study, could lose benefits and become detrimental. If this scenario were indeed the case it would be discouraging for therapeutic development as titrating the dose of EphA4 reduction in individuals would represent a challenge. While beneficial effects were seen in SOD1^G93A^ mice treated with an EphA4 ectodomain fused to Fc approach for inhibiting EphA4^8^, the complexities of multiple ephrin ligands that are shared between EphA4 and other ephrin receptors as well as of potential differential effects of forward and reverse ephrin-EphA receptor signaling make it hard to confirm a role for EphA4 inhibition in that study. Complexities about how to target EphA4 are also raised by the report that a compound acting as an agonist when tested in in vitro assays prolongs survival in SOD1^G93A^ mice^7^. Notably that study, which rigorously characterized EphA4 ligands, found no binding of the putative EphA4 inhibitors, compound 1, and rhynchophylline to EphA4 receptors. This calls into question the interpretations of previous experiments concluding a beneficial role for EphA4 in other neurodegeneration models that included some experiments using these compounds^2, 3^.

In broadly considering EphA4 as a therapeutic target, the results from preclinical SOD1 mouse model studies, which are equivocal at best, should be considered in the context of other available evidence. In the original identification of EphA4 as a disease modifier in ALS, in addition to preclinical model data, evidence for a correlation of blood EphA4 expression levels with age of ALS onset in patients was reported^2^. In addition, two examples of loss-of-function mutations in EphA4 found in ALS patients with unusually long survival were reported^2^. A recent study found that while the level of EphA4 mRNA in blood is weakly correlated with age of disease onset, it is not a significant predictor of disease progression^9^. In addition, recent rare variant and common variant analyses found no evidence that EphA4 is associated with ALS^17,18^. Overall, our results with inducible EphA4 KO in SOD1^G93A^ mice, despite caveats due to limitations of the model, when taken along with a growing body of evidence, raise significant concerns for the therapeutic hypothesis that EphA4 inhibition would be beneficial in ALS.

## Methods

### Mice

SOD1^G93A^ high copy number transgenic mice and their non-transgenic littermates were originally derived from Jackson Laboratory (Bar Harbor, ME; stock #002726) (Gurney, 1994) and were backcrossed at Genentech for > 20 generations into C57BL/6N (Charles River). Efforts were made to adhere to guidelines for preclinical research in ALS^19^, including ensuring that control groups had consistent strain, transgene, and copy number as experimental groups. Copy number was assessed as previously described^16^ and all SOD1^G93A^ animals used in the study fell into the expected high copy number range. Tamoxifen-dependent cre mice (CAG-CreERT) were generated as described previously^20^. EphA4flox mice (Jax 012916) were crossed with CAG-CreERT mice (JAX #004453) to generate EphA4flox;Cre positive or EphA4flox;Cre negative mice. These mice were then crossed with the SOD1^G93A^ mice generating, for example, SOD1^G93A^; EphA4flox; Cre negative and SOD1^G93A^; EphA4flox; Cre positive mice, which are referred to as SOD1^G93A^; EphA4WT and SOD1^G93A^; EphA4cKO, respectively. EphA4 excision was achieved by placing the mice on a tamoxifen-containing diet (about 40 mg/kg per day) for 4 weeks to induce Cre-mediated DNA recombination. In pilot studies this protocol was found to generate maximal recombination efficiency of the EphA4 allele in the CNS while minimizing the loss of animals due to tamoxifen toxicity. All mice in this study were placed on this diet, including non-Tg controls, where applicable. While on medicated chow, all mice appeared healthy and active overall despite modest weight loss (up to 10 to 15%). All mice were returned to regular chow diet (LabDiet 5010) after the tamoxifen treatment.

### qPCR measurement of EphA4 levels

Lumbar spinal cord and brain tissues were isolated from Cre-positive mice maintained on a normal diet with and without tamoxifen, and rapidly placed in eppendorf tubes containing RNALater (Ambien). Tissues were then transferred to Qiazol (Qiagen, RNA extraction kit) lysis reagent and lysed using a TissueLyser (Qiagen). RNAs were extracted using standard Qiagen RNeasy Lipid Tissue kit protocols. cDNA was generated using the High-capacity RT kit from Thermo Fisher Scientific, followed by qPCR assays on an Applied Biosystems ViiA7 thermal cycler using TaqMan expression assays for EphA4 (Mm00433056_m1) and Gapdh (Mm99999915_g1), obtained from Thermo Fisher Scientific.

### Study design

Mice were housed on a regular light/dark cycle (14:10 h) with ad libitum access to food and water. All behavioral assessments were conducted during the light phase. A hemizygous breeding design was used to produce littermate mice from all four genotypes. The studies included three cohorts of animals, which differ based on the age at when EphA4 knockdown was induced. Cohort 1 was placed on tamoxifen food at 4 weeks of age, cohort 2, at 7 weeks of age and cohort 3, at 17 weeks of age. Animals with in each cohort were group-housed across genotypes and cohorts for behavior were age and gender matched. All behavioral tests were performed by observers blinded to genotype. All protocols for mouse experiments were approved by the Institutional Animal Care and Use Committee and were conducted in accordance with the NIH Guide for the Care and Use of Laboratory Animals.

### Behavioral assessments

#### Body weight

Weekly body weights were recorded starting from the day before tamoxifen induction until take down.

### Compound muscle action potential (CMAP) measurement

CMAP amplitudes were assessed every two weeks. Mice were anesthetized with 2.5% isoflurane, right leg and hip were shaved and remaining hair was removed. Two stimulating needle electrodes were inserted perpendicular to the nerve into either side of the right sciatic notch. Recording needle electrodes were inserted into the Achilles tendon (anode) and into either the tibialis anterior (TA) or the gastrocnemius muscle (GA), and a digital ring ground electrode, coated in electrode cream, was placed on the mouse’s tail. Data was amplified (BioAmp, ADinstruments) and acquired with a sampling rate of 10 kHz, and filtered at 1 Hz high pass and 5 kHz low pass (Powerlab 4/25, ADInstruments). A controlled stimulus, with a pulse duration of 0.2 ms, was applied to the nerve to evoke contractions from either the Gastrocnemius (GA) or the Tibialis Anterior (TA) muscle in 2 mA increments, starting from 2 to as high as 50 mA, until the amplitude no longer increased. The maximum main wave amplitude (in mV) was measure from baseline to peak and was recorded for each mouse.

### Grip strength measurement

Grip strength was measured every two weeks and never on the same week that CMAP recording occurred. Grams of force were measured using dual sensor model Chatillon Grip Strength meters with digital force transducers (Columbus Instruments). For fore-paw measurements mice were suspended by the tail and allowed to grasp the pull bar. Once both paws were secured the mouse was pulled back along a straight line, by the tail, until it released its grip and maximum force was attained. For the hind-paw measurement mice were allowed to grasp a grip grid held by the testers right hand to stabilize the fore-paws, the tail was grasped by the left hand and the mouse’s hind paws were placed on the push bar. Once both paws were secure, the mouse was pulled back towards transducer in one fluid motion by tester and maximal force was obtained. Six consecutives measurements were recorded alternating between the fore and hind paws, such that there were three recordings per paw for each mouse. Data was plotted as an average of three trials for each paw.

### Tissue harvest and preparation

Mice were euthanized using 2.5% tribromoethanol (0.5 ml/25 g body weight) and transcardially exsanguinated with phosphate-buffered saline (PBS), followed by 4% paraformaldehyde (PFA) in PBS for fixation. Tibialis Anterior and Gastrocnemius (soleus removed) were stored in PBS at 4 degrees Celsius. On the day of shipment tibialis anterior muscles were submerged into a sucrose gradient: 10% sucrose for 2 h, 20% sucrose for 2–4 h and lastly, 30% sucrose and shipped overnight to Clarapath for sectioning and histology. Spinal cords were dissected and post-fixed overnight in 4% PFA, then transferred into PBS and shipped to NeuroScience Associates for sectioning and histology.

### Immunohistochemistry (IHC) for motor neurons and neuro muscular junctions (NMJ)

Lumbar spinal cords were sectioned and stained as described^14^. Tissues were embedded into a gelatin matrix using MultiBrain Technology (NeuroScience Associates) and each block was sectioned coronally at a thickness of 25 μm. A series of 33 sections, equally spaced at 300 mm intervals throughout the entire lumbar cord was used for staining. IHC staining was performed using goat anti-ChAT (choline acetyltransferase; Millipore AB144P) and rabbit anti-Iba1 (Wako 019-19741). Whole slide imaging was performed at 200 × magnification using the Nanozoomer (Hamamatsu Corp, San Jose, CA) system. 7–8 images per mouse in the L3-5 regions were analyzed using Matlab (Mathworks, Natick, MA). Tissue sections, motor neurons and microglia were detected using color thresholds and morphological operations. Regional minima and radial symmetry detection with watershed segmentation followed by morphological, shape and size filtering were used to further enumerate DAB positive cells. Image analysis was performed while blinded to experimental group and genotype.

NMJ sectioning, staining and analysis was conducted by Clarapath, Inc (New York, NY). Tibialis Anterior muscles were longitudinally sectioned at 20 μm. Slides were stained with Rabbit anti-VACHT (Life Technologies) and TMR-conjugated α-Bungarotoxin (Life Technologies). All stained slides were imaged using a 20 × objective (0.75 NA), at a resolution of 0.45 μm/pixel. A single focal plane was collected for all samples. Sections were analyzed to determine the total number of VACHT and TMR-α-bungarotoxin counts per muscle along with the percent VACHT/TMR-α-bungarotoxin per sample area. NMJs with 0–40% co-localization of staining were considered denervated. Image analysis was performed blinded to experimental grouping and genotype.

### Statistics

Statistical analyses were performed using Graph pad Prism v7.0 software (GraphPad Software Inc.) or JMP 14.2.0 software (SAS Institute Inc.). For measurements of phenotypes over time, a two-way repeat measures ANOVA (RMANOVA) was first conducted with factors genotype and time. When significant overall effects of genotype were observed, follow up analysis was performed with post hoc RMANOVA using full factorial analysis of EphA4 and SOD1 genotypes. A one-way ANOVA followed by Tukey’s T-test was used to test for effects of multiple genotypes at a single time point. For comparisons of only two genotypes at a single time point, a t-test was used. $ denotes significance compared to control (WT, EphA4WT) and * denotes significance for indicated comparisons, $ or * when *P* < 0.05, $$ or ** when *P* < 0.01, $$$ or *** when *P* < 0.001 and $$$$ or **** when P < 0.0001. All data are presented as means ± SEM.

## References

[1] Dion PA, Daoud H, Rouleau GA (2009). Genetics of motor neuron disorders: New insights into pathogenic mechanisms. Nat. Rev. Genet..

[2] Van Hoecke A (2012). EPHA4 is a disease modifier of amyotrophic lateral sclerosis in animal models and in humans. Nat. Med..

[3] Fu AK (2014). Blockade of EphA4 signaling ameliorates hippocampal synaptic dysfunctions in mouse models of Alzheimer's disease. Proc. Natl. Acad. Sci. U S A.

[4] Vargas LM (2014). EphA4 activation of c-Abl mediates synaptic loss and LTP blockade caused by amyloid-beta oligomers. PLoS ONE.

[5] Goldshmit Y, Galea MP, Wise G, Bartlett PF, Turnley AM (2004). Axonal regeneration and lack of astrocytic gliosis in EphA4-deficient mice. J. Neurosci..

[6] Lemmens R, Jaspers T, Robberecht W, Thijs VN (2013). Modifying expression of EphA4 and its downstream targets improves functional recovery after stroke. Hum. Mol. Genet..

[7] Wu B (2017). Potent and selective EphA4 agonists for the treatment of ALS. Cell Chem. Biol..

[8] Zhao J, Cooper LT, Boyd AW, Bartlett PF (2018). Decreased signalling of EphA4 improves functional performance and motor neuron survival in the SOD1(G93A) ALS mouse model. Sci. Rep..

[9] Ling KK (2018). Antisense-mediated reduction of EphA4 in the adult CNS does not improve the function of mice with amyotrophic lateral sclerosis. Neurobiol. Dis..

[10] Rue L (2019). Reducing EphA4 before disease onset does not affect survival in a mouse model of amyotrophic lateral sclerosis. Sci. Rep..

[11] Dottori M (1998). EphA4 (Sek1) receptor tyrosine kinase is required for the development of the corticospinal tract. Proc. Natl. Acad. Sci. U S A.

[12] Lai KO, Ip FC, Cheung J, Fu AK, Ip NY (2001). Expression of Eph receptors in skeletal muscle and their localization at the neuromuscular junction. Mol. Cell Neurosci..

[13] Deitch JS (2014). Phenotype of transgenic mice carrying a very low copy number of the mutant human G93A superoxide dismutase-1 gene associated with amyotrophic lateral sclerosis. PLoS ONE.

[14] Le Pichon CE (2013). EGFR inhibitor erlotinib delays disease progression but does not extend survival in the SOD1 mouse model of ALS. PLoS ONE.

[15] Le Pichon, C. E. *et al.* Loss of dual leucine zipper kinase signaling is protective in animal models of neurodegenerative disease. *Sci. Transl. Med.* **9**, 10.1126/scitranslmed.aag0394 (2017). 10.1126/scitranslmed.aag0394 28814543

[16] Sengupta-Ghosh A (2019). Muscle specific kinase (MuSK) activation preserves neuromuscular junctions in the diaphragm but is not sufficient to provide a functional benefit in the SOD1(G93A) mouse model of ALS. Neurobiol. Dis..

[17] Gaastra B (2016). Rare genetic variation in UNC13A may modify survival in amyotrophic lateral sclerosis. Amyotroph. Lateral Scler. Frontotemporal Degener..

[18] van Rheenen, W. *et al.* Genome-wide association analyses identify new risk variants and the genetic architecture of amyotrophic lateral sclerosis. *Nat. Genet.* **48**, 1043–1048, 10.1038/ng.3622 (2016). 10.1038/ng.3622 PMC5556360 27455348

[19] Ludolph AC (2010). Guidelines for preclinical animal research in ALS/MND: A consensus meeting. Amyotroph. Lateral Scler..

[20] Pozniak CD (2013). Dual leucine zipper kinase is required for excitotoxicity-induced neuronal degeneration. J. Exp. Med..

